# Construct validity and responsiveness of the simplified version of Ankylosing Spondylitis Disease Activity Score (SASDAS) for the evaluation of disease activity in axial spondyloarthritis

**DOI:** 10.1186/s12955-014-0129-9

**Published:** 2014-08-22

**Authors:** Fausto Salaffi, Alessandro Ciapetti, Marina Carotti, Stefania Gasparini, Gustavo Citera, Marwin Gutierrez

**Affiliations:** Rheumatology Department, Polytechnic University of the Marche, Jesi, Ancona Italy; Radiology Department, Polytechnic University of the Marche, Ospedali Riuniti di Ancona, Via Conca 71, 60126 Ancona, Italy; Section of Rheumatology, Instituto de Rehabilitacion Psicofisica, Calle Echeverria, 955 Buenos Aires, Argentina

**Keywords:** Axial-SpA, SASDAS, ASDAS, BASDAI, Disease activity

## Abstract

**Background:**

Over the last decade, significant progresses have been achieved in the development and validation of new tools for the evaluation of disease activity in axial spondyloarthritis (SpA). Despite they play a key role in the assessment of these patients, the calculation scores are relatively complex and difficult to be quickly assessed in the busy daily clinical practice.

**Objectives:**

To test the construct validity of the Simplified Ankylosing Spondylitis Disease Activity Score (SADSAS) to define disease activity and compare its internal and external responsiveness with the Ankylosing Spondylitis Disease Activity Score (ASDAS) and the Bath Ankylosing Spondylitis Disease Activity Index (BASDAI) in patients with axial SpA.

**Methods:**

The patient cohort comprised 397 consecutive axial SpA patients who had never been treated with tumor necrosis factor (TNF) blockers. Clinical and laboratory outcome assessments were performed at baseline, and at week 24. The following parameters were evaluated: BASDAI, ASDAS-CRP, ASDAS-ESR, and SASDAS. Construct convergent validity was evaluated by correlating SASDAS with ASDAS CRP/ESR, BASDAI, Bath Ankylosing Spondylitis Functional Index (BASFI) and EuroQol five-dimensional (EQ-5D) questionnaire. One hundred and fifty-six patients were observed longitudinally for 6 months. Responsiveness was assessed after six months of treatment with sulfasalazine (SSZ) or biologics. Internal responsiveness was evaluated by using the effect size (ES) and standardized response mean (SRM). External responsiveness was investigated by receiver operating characteristic (ROC) analysis. Change scores were compared by calculating paired *t*-test statistic for the difference.

**Results:**

In testing for convergent validity a strong correlations (p < 0.0001) were observed between both SASDAS and ASDAS-ESR (r = 0.835), and ASDAS-CRP (r = 0.805). Strong correlations (p < 0.0001) were also found between SASDAS and BASDAI score (r = −0.886), SASDAS and BASFI scores (rho = 0.588) and SASDAS and EQ-5D scores (rho = −0.579). The cross-classification showed a significant overall agreement (defined as the percentage of observed exact agreements) for SASDAS vs ASDAS-ESR (weighted k = 0.704) and for SASDAS vs ASDAS-CRP (k = 0.661). The most efficient composite measure in detecting change was the ASDAS-CRP (ES 1.95 and SRM 0.97). The responsiveness of SASDAS was slightly higher to ASDAS-ESR with an ES of 1.62 and 1.33, and an SRM of 0.88 and 0.71, respectively. The BASDAI appear to be the less responsive (ES = 0.93 and SRM = 0.52). The area under ROC curve of the SASDAS gives similar results to those provided by ASDAS CRP/ESR. The score changes of all combinations were highly correlated (p < 0.0001).

**Conclusions:**

The new SASDAS is a highly effective measure in assessing disease activity and it showed comparable internal and external responsiveness with respect to the ASDAS ESR/CRP response criteria in patients with axial SpA. SASDAS is easy to calculate and, therefore, appear suitable for clinical decision making, epidemiologic research, and clinical trials.

**Electronic supplementary material:**

The online version of this article (doi:10.1186/s12955-014-0129-9) contains supplementary material, which is available to authorized users.

## Background

Axial spondyloarthritis (SpA) include diseases with predominantly axial involvement, such as ankylosing spondylitis (AS), psoriatic arthritis (PsA) and non-radiographic axial SpA which have as key symptoms both inflammatory back pain and stiffness [1–4].

Over the last decade, significant advances have been achieved in the development and validation of new tools for the evaluation of disease activity in axial SpA [5]. Most of them are based on self-reported questionnaires that include evaluation of pain and stiffness, patient’s or physician’s global assessment (PtGA or PhGA, respectively), acute phase reactants evaluation or on the Bath Ankylosing Spondylitis Disease Activity Index (BASDAI) [6] which is most frequently used in clinical trials. Although BASDAI has been endorsed by ASAS for the treatment monitoring and measurement of disease activity in axial SpA [7], it demonstrated to have a limited face and construct validity. Moreover it is not sensitive to change (lack of responsivity) [8] and does not include any objective measures of activity [9]. Recently, AS Disease Activity Score (ASDAS) has been proposed by ASAS working Group for the evaluation of disease activity in patients with AS [10,11]. ASDAS is the first validated disease activity system which combines both patient-reported outcome measures and acute-phase reactants levels. However, the equation used to calculate the ASDAS score is relatively complex (since requires a calculator) to be quickly assessed in the busy daily clinical practice. In this way Sommerfleck et al. [12] developed a simplified version of the ASDAS, named Simplified AS Disease Activity Score (SASDAS) which, keeping the sensitive characteristics of the ASDAS, can be considered an intuitive and easy way to assess the disease activity in patients with axial SpA. SASDAS is based on the recently developed disease activity indices for rheumatoid arthritis (RA) such as the Simplified Disease Activity Index (SDAI) [13] and disease activity index for the assessment of reactive arthritis (DAREA) [14] which demonstrated to be valid and reliable in daily clinical practice in AS patients.

Taking into account these information we addressed the aims of our study in the following points: 1) to test the construct validity of the SASDAS to define disease activity in patients with axial SpA and 2) to compare its internal and external responsiveness with ASDAS CRP/ESR and the BASDAI, in an observational cohort of patients with axial SpA.

## Methods

### Patient characteristics

The investigated cohort included 397 consecutive axial SpA patients (298 men, 99 women; range 19–78 years old, mean age 53.4 years old) with disease duration of 5.1 years (SD 11.8). The classification of axial SpA was based on fulfilment of the ASAS classification criteria that are defined as follows: the presence of sacroiliitis by radiography or by magnetic resonance imaging (MRI) plus at least one SpA feature (“imaging arm”) or the presence of HLA-B27 plus at least two SpA features (“clinical arm”) [3,4]. Radiographs were scored for sacroiliitis according to the modified New York criteria [15] (defined as grade I: some blurring of the joint margins – suspicious, grade II - minimal sclerosis with some erosion, grade III: definite sclerosis on both sides of joint - severe erosions with widening of joint space with or without ankylosis, grade IV: complete ankylosis).

The anatomical region of the axial skeleton evaluated by MRI was chosen by both rheumatologist and radiologist after consensus, according to the patient’s symptoms and including always the sacroiliac joints [16–18]. MRI of the sacroiliac joints was performed in 31 patients. MRI of the sacroiliac joints plus the spine was performed in further 9 patients. Patients with peripheral arthritis were excluded by our study. Peripheral arthritis was considered in presence of clinical tender and swollen joints assessed by a rheumatologist. Polyarthritis was defined as five or more inflamed (swollen or tender) joints as suggested by Helliwell et al. [19]. Further exclusion criteria were the following: other active concomitant musculoskeletal diseases (e.g. gout or CPPD, rheumatoid arthritis), history of cancer or lymphoproliferative disease, uncontrolled diabetes, unstable ischemic heart disease, congestive heart failure, active inflammatory bowel disease, positive serology for hepatitis B, history of active tuberculosis and concomitant fibromyalgia [20]. All patients were treated with non-steroidal antiinflammatory drugs on an on-demand basis and 77 patients (19.4%) were taking low-dose of corticosteroids (mean 4.6 mg/day of prednisolone, range 2.5–16 4.6 mg/day).

One-hundred and fifty-six patients (119 women, 37 men; range 19–76 years old, mean age 54.6 years old), were followed for 6 months. Considering that it was not a randomised trial, drug therapy was chosen by the managing clinician as considered the most appropriate [21,22].

At baseline, 29 patients were already treated with sulfasalazine (SSZ) previously commenced in primary care (25 patients) or in gastroenterology setting (4 patients affected by inflammatory bowel disease). The dosage of SSZ was 2.0 g/day or up to 3.0 g/day depending on the efficacy and tolerance. In 21 of 29 SSZ was replaced with anti-TNF blockers within the third month after the recruitment. The remaining 8 patients continued treatment with non-steroidal antinflammatory drugs (NSAIDs) administered periodically. A total 127 patients were on TNF-blockers (81.4%), including infliximab (29.9%), etanercept (33.1%) and adalimumab (37%). Infliximab (5 mg/kg) was given intravenously at baseline and after two and six weeks and, by then, every eight weeks. In case of inadequate response, the frequency of infliximab treatment was raised to every six weeks. Etanercept was administered as a subcutaneous injection once (50 mg) or twice (25 mg) a week. Adalimumab (40 mg) was administered as a subcutaneous injection on alternate weeks. The choice of the anti TNF agent was based on the judgment of the rheumatologist and/or on the specific needs of the patient. Patients were allowed to receive concomitant medication as usual in daily clinical practice.

All patients were attending the outpatient and inpatient clinics of the Rheumatology Department of the Università Politecnica delle Marche (Ancona, Italy) and they represent a “real life” sample of axial SpA. The study was approved by the Hospital Clinic ethics committee. All patients agreed to be enrolled in the study and signed informed consent.

### Measures of disease activity

Clinical and laboratory outcome assessments were performed at baseline and after 24 week and include the evaluation of BASDAI, ASDAS based on ASDAS-CRP or ASDAS-ESR and SASDAS indices. The ESR (mm/hour) and CRP serum levels (mg/dl) were also collected. The BASDAI contains six items representative of disease activity in AS [23]. Each item is provided of a 10‐cm horizontal numerical rating scale (NRS) anchored by adjectival descriptors ‘none’ and ‘very severe’. Item 6 (morning stiffness, duration) is anchored by a time scale (0–2 h). The mean of items 5 (morning stiffness, severity) and 6 is calculated. The total score is converted to a 0–10 scale, with a lower score indicating lower disease activity. A cutoff level of 4 is used to define the presence of an active disease [24]. Usually, patients understand and prefer NRS more than visual analogue scale (VAS) [25,26].

ASDAS is a composite score of disease activity comprising three items from BASDAI (1) back pain (question 2), (2) peripheral pain/swelling (question 3) and (3) duration of morning stiffness (question 6), and patient’s global assessment and CRP. The development studies resulted in four candidate ASDAS scores, that fulfilling the clinimetric properties of truth, feasibility and discrimination. The membership has selected the ASDAS with CRP as the preferred version and with ESR as the alternative version [10,11]. The ASDAS formulas are the following:

- *ASDAS* − *CRP* = 0.121 ∗ *backpain* + 0.058 ∗ *duration of morning stiffness* + 0.110 ∗ *patient* ' *s global assessment* + 0.073 ∗ *peripheral pain*/*swelling* + 0.579 ∗ *Ln*(*CRP* + 1).
- *ASDAS* − *ESR* = 0.079 ∗ *back pain* + 0.069 ∗ *duration of morning stiffness* + 0.113 ∗ *patient's global assessment* + 0.086 ∗ *peripheral pain*/*swelling* + 0.293 ∗ √ (*ESR*).
- CRP is in mg/litre, ESR is in mm/h; the range of other variables is from 0 to 10; Ln represents the natural logarithm; √ represents the square root.

The ASDAS has been validated and found to be discriminatory in assessing disease activity in axial SpA and it has been endorsed by the ASAS and by Outcome Measures in Rheumatology (OMERACT) [27]. The published cut-offs of ASDAS are the following: <1.3 for inactive disease, <2.1 for moderate disease activity, <3.5 for high disease activity, and ≥3.5 for very high disease activity. An improvement of ≥ 1.1 units is considered as a clinical significant improvement and an improvement of two units is considered as a major response [27,28]. SASDAS was calculated by the simple linear addition of ASDAS which includes five components: patient global assessment (NRS 0–10 cm), back pain (BASDAI question no. 2), peripheral pain and swelling (BASDAI question no. 3), duration of morning stiffness (BASDAI question no. 6), and ESR in millimeters per hour, divided by 10. The cut-off values for SASDAS were the following: inactive disease from 0 to 7.8, moderate disease activity from 7.9 to 13.8, high disease activity from 13.9 to 27.6 and very high activity above 27.6 [12].

### Functional limitation and health status assessments

Functional limitation and health status assessments were performed at baseline and include an evaluation of Bath Ankylosing Spondylitis Functional Index (BASFI) [29] and EuroQol five-dimensional (EQ-5D) questionnaire [30]. The BASFI consists of 10 questions designed to determine the degree of functional limitation in patients with AS. Each question is answered using an 11-numbered button NRS format, with a recall period of the past week. The mean of the 10 scales affords the BASFI score - a value between 0 and 10, with a lower score indicating less functional limitation [29]. The paper formats in the Italian language of the BASFI and the BASDAI indices, previously validated were employed in this study [31]. The EQ-5D health state classifier consists of 5 single-item dimensions - mobility, self-care, usual activities, pain/discomfort, and anxiety/depression - with 3 levels of response for no, some, or extreme problems in each dimension [30]. In addition to the health state classifier, patients rated their current health on a 20-cm visual analog scale (EQ-5D VAS) ranging from 0 (worst possible health state) to 100 (best possible health state). Responses to these five dimensions are converted into one of 243 different EQ-5D health state descriptions, which range between no problems on all five dimensions (11111) and severe/extreme problems on all five dimensions (33333). The Italian population-based values were used to convert patient responses to the health state classifier into a single index, which produces scores from 1 to −0.38 [32].

#### Statistical analysis

Data related to composite indices and BASDAI showed a parametric distribution (tested with the Kolmogorov–Smirnov test) and were presented as means with standard deviations (SDs). Whereas BASFI and EQ-5D showed a non-normal distribution (tested with the Kolmogorov–Smirnov test). Overall agreement (defined as the percentage of observed exact agreements) of SASDAS and different cut-off ASDAS ESR/CRP activity states were calculated by weigthted Cohen’s kappa coefficients. Evidence for construct validity can only be accumulated by ‘a priori’ hypothesized patterns of associations with other validated instruments. In this study, the construct validity of the SASDAS was examined in two ways. First, we examined construct convergent validity by correlating the scores of the SASDAS index with ASDAS CRP/ESR, BASDAI, BASFI and EQ-5D. A specific subscale is expected to converge with the scores of those instruments targeting the same construct and to deviate from the scores given by instruments or scales assessing a different one (divergent validity). To quantify these relationships, Pearson’s correlation coefficient and Spearman’s rho correlation coefficients were obtained. Correlations > 0.90 were interpreted as very high, 0.70–0.89 as high, 0.50–0.69 as moderate, 0.26–0.49 as low and ≤ 0.25 as little if any correlation occurred. Furthermore, we have created patient groups based on the patients’ activity ranks within the cohort and used Cohen’s weighted Kappa coefficients to assess the level of agreement of different activity categories on individual patients. For this purpose, the ASDAS cut-off scores were categorised into 4 groups [28]. Similarly, the SASDAS scores were categorised into 4 groups as follows: from 0 to 7.8 (inactive disease), from 7.9 to 13.8 (moderate disease activity), from 13.9 to 27.6 (high disease activity), and above 27.6 (very high activity) [12]. Responsiveness was evaluated by longitudinal assessment of patients, investigating if the measures were sensitive to change following the intervention. Responsiveness refers to the ability of an elicitation method to accurately detect a meaningful change over time when it has occurred. In accordance with Husted et al. [33], we distinguished between internal and external responsiveness. Internal responsiveness refers to the ability of a measure to change over a pre-established time frame, whereas external responsiveness describes the relationship between changes in a measurement and changes in a reference measure of disease activity. To assess the magnitude of the internal responsiveness, we have calculated the effect size (ES) and standardized response mean (SRM) [34]. The ES is defined as the mean change in the score between baseline and follow-up, which is divided by the SD of the baseline score. The SRM is defined as the mean change in the scores between baseline and follow-up which is divided by the SD of the individual changes in the scores. Higher values of ES or SRM mean greater responsiveness of the measure. Values ≤ 0.5, between 0.5 and 0.8, and ≥ 0.8 were considered to represent small, moderate and large degrees of responsiveness, respectively. Considering that each of these indices is sensitive to change for the declined group, we supplemented them by computing the paired samples *t*-test statistic for the difference in change scores. Change between baseline and 6-month follow-up assessments was considered significant when p < 0.05. External responsiveness was investigated with receiver operating characteristic (ROC) curve analysis in categories of respondents, stratified according to the response on an item on change in overall health during the past 6-months. We used item two of the SF-36 Health Survey (SF-36) questionnaire (“compared to 6-months ago, how would you rate your health in general now? (1 = much better, 2 = somewhat better, 3 = about the same, 4 = somewhat worse, 5 = much worse”) to rate the overall change. This method has the advantage of synthesizing information on the sensitivity and specificity for detecting improvement by an external criterion [34]. The area under the ROC curve (AUC-ROC) in this setting can be interpreted as the probability of correctly identifying the improved patients from non-improved patients. This area ranges from 0.5 (no accuracy in distinguishing improved from non-improved) to 1.0 (perfect accuracy). According to Swets [35] areas from 0.50 to 0.70 represent poor accuracy, those from 0.70 and 0.90 are useful for some purposes and higher values represent high accuracy. Since ROC analysis requires external criteria to be dichotomous, the categories of “about the same, somewhat worse” and “much worse” were collapsed to one variable (non-improved patients) for our analysis. The non-parametric Wilcoxon signed ranks test is used for calculation and comparison of the areas under the ROC curves derived from the sample of patients, as suggested by Hanley and McNeil [36]. All data were entered into a Microsoft Access database which was developed for the management of the cross-sectional study. All the statistical analyses were performed using the SPSS version 15.0 (SPSS Inc, Chicago, USA) and the MedCalc® version 11.0 (MedCalc Software, Mariakerke, Belgium).

## Results

Table 1, shows the demographic, laboratory and clinical data of the cohort of patients.

**Table 1:** **Demographic, laboratory and clinical characteristics of study population**

**Patients characteristics**	**Mean**	**SD**	**Median**	**25 - 75 P**
Age (years)	53.40	11.78	54.00	45.00 - 62.00
Disease duration (years)	5.13	4.95	4.00	2.00 - 7.00
ASDAS-ESR	2.48	0.65	2.49	2.06 - 2.93
ASDAS-CPR	2.56	0.77	2.53	2.04 - 3.13
SASDAS (0–10)	20.18	7.33	20.10	14.30 - 25.82
BASDAI (0–10)	4.10	1.48	4.00	3.15 - 5.12
• BASDAI item 1: fatigue/tiredness	6.00	2.34	6.00	4.00 - 8.00
• BASDAI item 2: neck back or hip pain	5.04	2.16	5.00	3.75 - 7.00
• BASDAI item 3: pain and swelling in other joints	4.88	2.65	5.00	3.00 - 7.00
• BASDAI item 4: discomfort from areas tender to touch	1.98	1.38	2.00	1.00 - 2.25
• BASDAI item 5: level of morning stiffness	3.73	1.83	3.50	2.50 - 5.00
• BASDAI item 6: duration of morning stiffness	3.61	2.24	3.00	2.00 - 5.00
BASFI (0–10)	4.47	1.99	4.44	3.20 - 5.55
EQ-5D score (0–1)	0.70	0.13	0.68	0.61 - 0.78
Patient’s global status (0–10)	5.05	2.38	5.00	4.00 - 7.00
CRP level, mg/l	9.19	9.28	6.10	3.30 - 11.44
ESR, mm/hour	17.4	10.51	16.50	8.40 - 20.80

*Abbreviations:* *(ASDAS)* Ankylosing Spondylitis Disease Activity Score, *(SADSAS)* Simplified Ankylosing Spondylitis Disease Activity Score, *(BASDAI)* Bath Ankylosing Spondylitis Disease Activity Index, *(BASFI)* Bath Ankylosing Spondylitis Functional Index, *(EQ-5D)* EuroQol five-dimensional questionnaire, *(ESR)* Erythrocyte Sedimentation Rarate, *(CRP)* C-Reactive Protein.

### Score distributions of the disease activity indices

**Figure 1:**
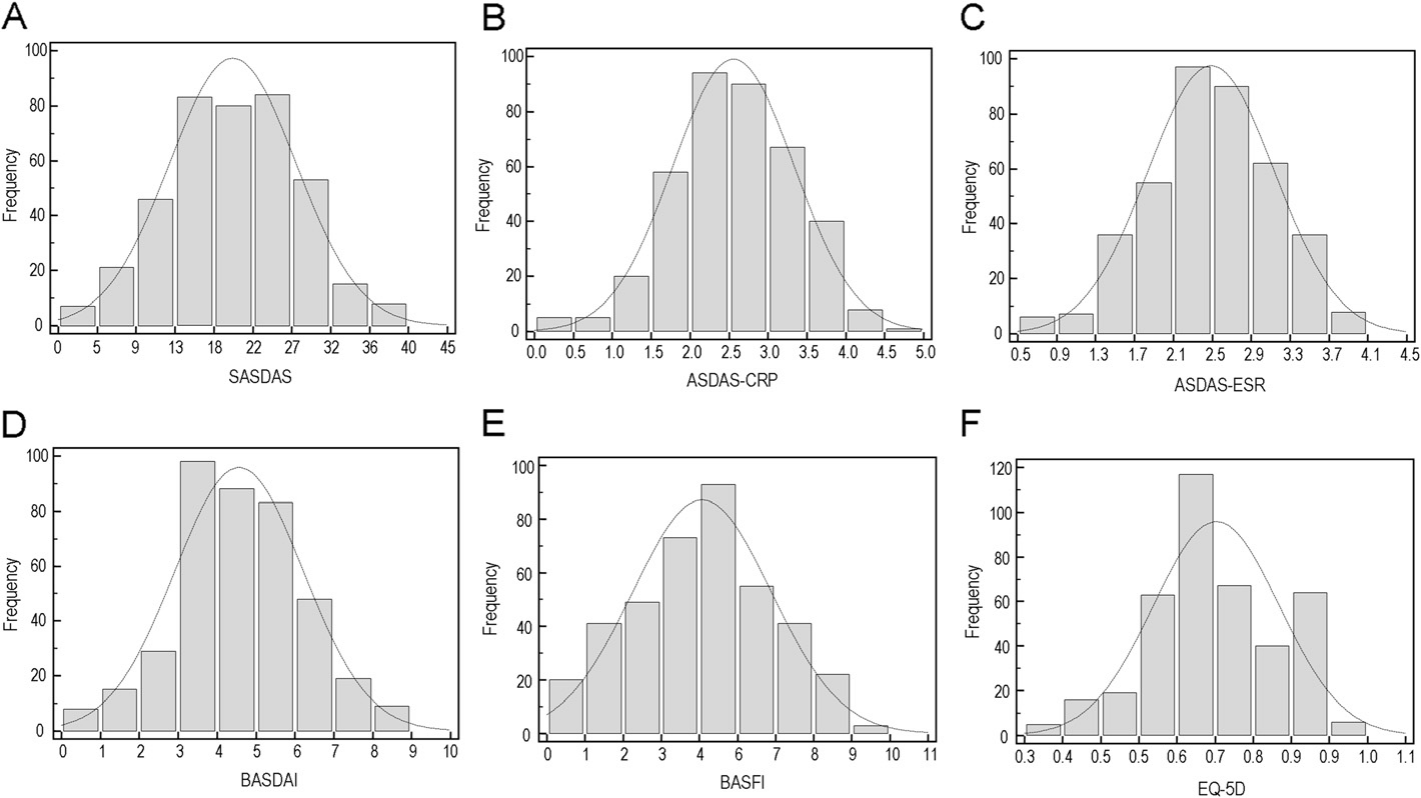
**Histograms demonstrating central-tendency estimation and distributions of SASDAS (A) and ASDAS ESR/CRP composite indices (B-C), BASDAI (D), BASFI (E) and EQ-5D (F).** The bar on the left of each group represents the number of subjects with a score of 0 (floor effect). The bar on the right represents the number of subjects with a maximum possible score (ceiling effect).

Additional file 1 summarizes the descriptive statistics for SASDAS and ASDAS ESR/CRP scores, BASDAI, BASFI and EQ-5D. Figure 1 presents estimates of central tendency and distribution of score for SASDAS (A), ASDAS ESR/CRP (B-C), BASDAI (D), BASFI (E) and EQ-5D (F) in all patient at baseline (N = 357 patient). The bar on the left of each graph represents the number of subjects with a score of 0 (floor effect); the bar on the right represents the number of subjects with a maximum possible score (ceiling effect). All activity indices were normally distributed whereas BASFI and EQ-5D showed a non-normal distribution. The mean (SD) were as follows: SASDAS 20.18 (7.33), ASDAS-ESR 2.48 (0.65), ASDAS-CRP 2.56 (0.77), BASDAI 4.10 (1.48), BASFI 4.47 (1.99) and EQ-5D 0.70 (0.13).

### Construct validity of the SASDAS in cross-sectional study

#### Concurrent validity

**Figure 2:**
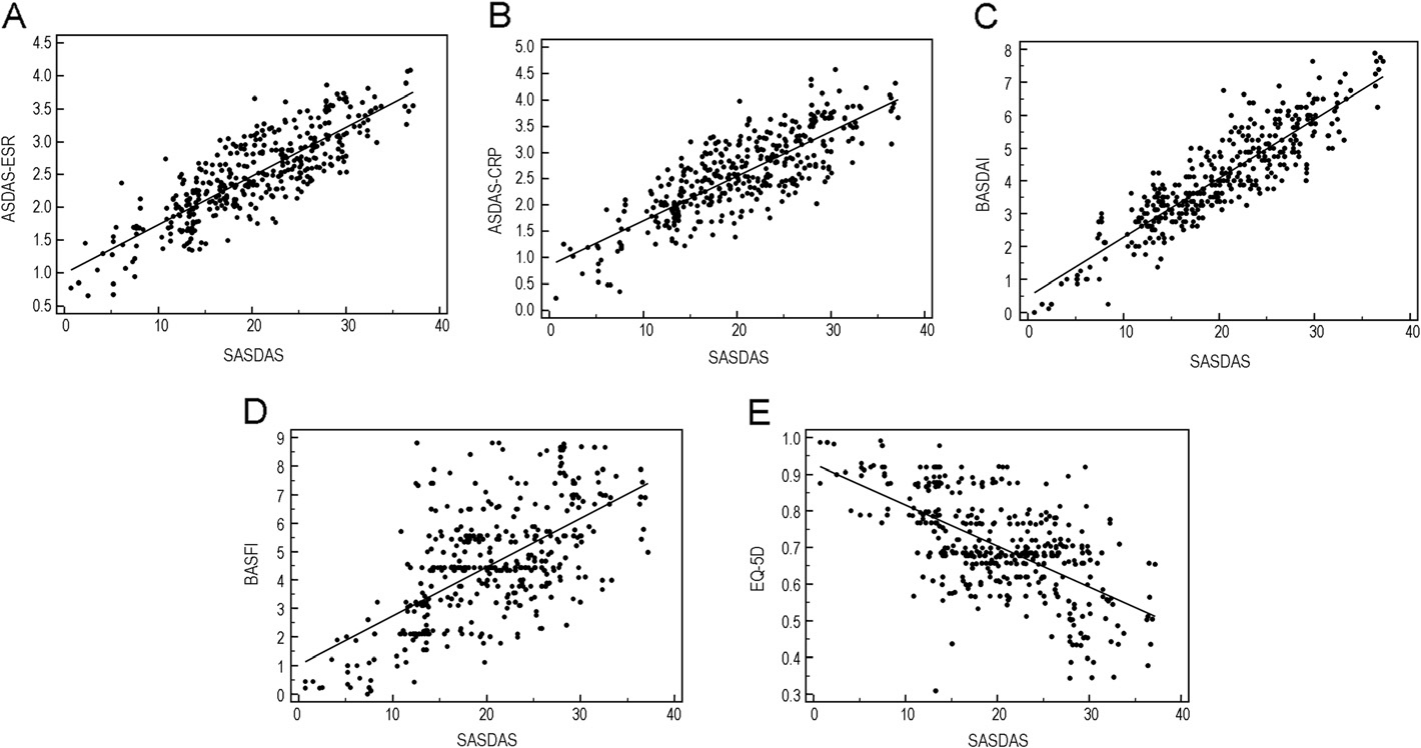
**Scatter plots of the composite disease activity indices at baseline.** All analyses indicate a highly significant degree (p < 0.0001) of correlation among the composite indices: **(A)** SASDAS versus ASDAS-ESR, **(B)** SASDAS versus ASDAS-CRP, **(C)** SASDAS versus BASDAI, **(D)** SASDAS versus BASFI, **(E)** SASDAS versus EQ-5D.

There was a very high degree of correlation between the composite indices. The indices were correlated significantly with all other comparator scores (p < 0.0001). The highest correlations were seen between SASDAS and ASDAS-ESR (r = 0.835) and between SASDAS and BASDAI score (r = −0.886). Strong correlations were also found between SASDAS and ASDAS-CRP (r = 0.805), SASDAS and BASFI (rho = 0.588) and SASDAS and EQ-5D (rho = −0.579) (Figure 2). The SASDAS showed no significant relationship with age and disease duration. Categorizing patients according to the proposed SASDAS disease activity scoring system revealed 20 patients (5.0%) with inactive disease, 84 patients (21.2%) with moderate disease activity, 246 patients (62.0%) with high disease activity and 47 patients (11.8%) with very high disease activity. According to the ASDAS-ESR, 24 patients (6.0%) had inactive disease, 64 patients (16.1%) moderate disease activity, 241 patients (60.7%) high disease activity and 68 patients (17.1%) very high disease activity. The cross-classification showed a significant agreement (weighted Kappa 0.704 with standard error of 0.038) (Table 2). The categorization of cut-off of SASDAS versus those of the ASDAS-CRP index have basically confirmed the agreement of the previous one (weighted Kappa 0.661 with standard error of 0.039) (Table 2).

**Table 2:** **Overall agreement (defined as the percentage of observed exact agreements) of SASDAS and ASDAS-ESR/CRP for different activity states in the axial SpA patient cohort at baseline (397 patients)**

		**Cut-off SASDAS**				
		**Very high**	**Hight**	**Moderate**	**Inactive**	**Total**
**Cut-off ASDAS-ESR**	Very high	44	24	0	0	68 (17.1%)
	High	3	212	26	0	241 (60.7%)
	Moderate	0	10	54	0	64 (16.1%)
	Inactive	0	0	4	20	24 (6.0%)
	Total (%)	47 (11.8%)	246 (62.0%)	84 (21.2%)	20 (5.0%)	397
**Cut-off ASDAS-CRP**	Very high	43	7	0	0	50 (12.6%)
	High	24	208	14	0	246 (62.0%)
	Moderate	1	26	50	5	82 (20.7%)
	Inactive	0	0	0	19	19 (4.8%)
	Total (%)	68 (17.1%)	241 (60.7%)	64 (16.6%)	24 (6.0%)	397

### a) Internal responsiveness

#### Effect size and standardized response mean statistics

All composite indices were responsive in detecting disease activity in the cohort of patients, with ES and SRM values higher observed from the BASDAI (Table 3). The most efficient composite measure in detecting change was the ASDAS-CRP (ES 1.95 and SRM 0.97). The least responsive in detecting change was the BASDAI (ES = 0.93 and SRM = 0.52). The responsiveness of SASDAS was slightly higher to ASDAS-ESR with an ES of 1.62 and 1.33, respectively and an SRM of 0.88 and 0.71. Inspection of ES reveals that this index gives the highest values.

**Table 3:** **Responsiveness statistics for SASDAS, ASDAS-ESR, ASDAS-CRP and BASDAI for patients with axial-SpA at 6-month follow-up (n = 157)**

	**Baseline mean values (SD)**	**Final mean values (SD)**	**Average change (SD)**	**Paired t test (p-value)**	**Effect size statistic (ES)**	**Standardized response mean (SRM)**
SASDAS	22.81 (5.19)	14.78 (7.07)	8.15 (9.23)	−10.98 (p < 0.0001)	1.62	0.88
ASDAS-ESR	2.60 (0.48)	2.01 (0.62)	0.60 (0.85)	−8.79 (p < 0.0001)	1.33	0.71
ASDAS-CRP	2.84 (0.52)	1.90 (0.73)	0.95 (0.97)	−12.16 (p < 0.0001)	1.95	0.97
BASDAI	4.45 (1.13)	3.69 (1.42)	0.71 (1.08)	−6.129 (p < 0.0001)	0.92	0.52

### b) External responsiveness

#### Receiver operating characteristic (ROC) curve analysis

**Figure 3:**
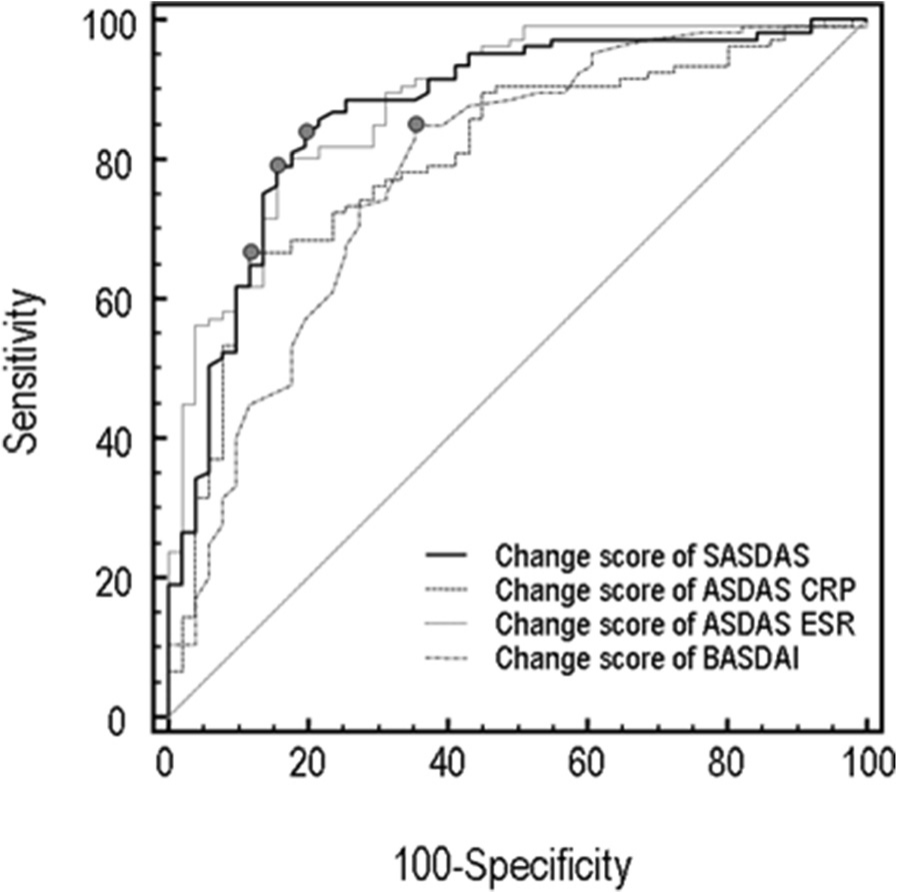
**ROC curves illustrating the relationship between sensitivity and complement of specificity (100 specificity) in axial SpA for the composite disease activity indices (ASDAS-ESR, ASDAS-CRP and SASDAS) and BASDAI, by using changes of global disease activity as external indicator.** The area under the ROC curve (AUC) can be interpreted as the probability of correctly identifying patients improved form those not-improved. A line that runs diagonally across the figure from lower left upper right will have an area of 0.5 which represent an instrument not able to discriminate different status of disease activity.

Figure 3 shows the ROC plots of changing scores of the three traditional composite disease activity indices and BASDAI, by using the item two on the SF-36 questionnaire to rate the overall change as an external criterion. For SASDAS, ASDAS-ESR, and ASDAS-CRP the AUC were 0.870 ± 0.031 (95% C.I. from 0.808 to 0.932), 0.794 ± 0.037 (95% C.I. from 0.721 to 0.867) and 0.882 ± 0.028 (95% C.I. from 0.826 to 0.937), respectively. Concerning the ROC plots of the change score of questionnaire, the AUC for BASDAI AUC was 0.787 ± 0.041 (95% C.I. from 0.704 to 0.868) (Additional file 2). The difference between changing scores of BASDAI and both SASDAS and ASDAS-CRP were significant (differences between areas = 0.085 ± 0.038 with 95% CI 0.009–0.161; p = 0.026 and 0.090 ± 0.043 with 95% CI 0.013–0.182; p =0.022, respectively).

#### Comparison of score changes by longitudinal analysis

**Figure 4:**
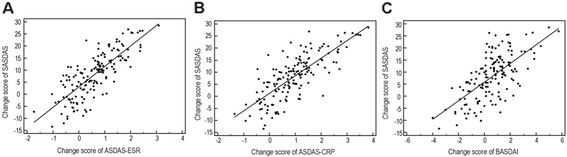
**Scatter plot of patient data showing the change in SASDAS compared with the change score of ASDAS-ESR (A), ASDAS-CRP (B) and BASDAI (C) at 6 months of follow-up.** Changes of SASDAS were all significantly (p < 0.0001), related to changes of ASDAS-ESR ASDAS-CRP and BASDAI, in response to treatment (*r* = 0.784; *r* = 0.774 and r = 0.807).

To further investigate the external responsiveness changing scores of composite disease activity indices were compared by calculating correlation coefficients. The changing scores of all combinations were highly correlated (p < 0.0001) (Figure 4). In particular, there was a strong correlations between mean change of the ASDAS-ESR score with changes of the SASDAS (r = 0.784, p < 0.0001) (A) and between mean change of the ASDAS-CRP and SASDAS score (r = 0.774, p < 0.0001) (B). Similarly, we have found a significant, but lower correlation, between mean change in the SASDAS score with mean changes in the BASDAI (r = 0.660, p < 0.0001) (C).

## Discussion

Several recent studies have been performed to identify and measure the outcomes of treatment of axial SpA in both research and clinical practice. The development of valid, reproducible and objective tool for the evaluation of disease activity in axial SpA is difficult, although valuable instruments have been recommended by several researchers [37]. Among the proposed composite indices, BASDAI has acceptable properties as a measure of disease activity in axial SpA. Nevertheless, there are few issues with regard the items content and appropriateness of response formats of BASDAI [38]. Further, it has been shown that the BASDAI is an ambiguous measure of disease activity in patients with peripheral or axial disease activity and that reflects only patient’s perspectives and not necessarily captures the entire spectrum of disease activity [39]. For that reason ASAS group has tried to go a step further in the evaluation of disease activity in AS by developing the ASAS-endorsed disease activity score (termed ASDAS) [10]. The ASDAS is an index that tries to reflect several aspects of disease activity and correlates well with both physician’s and patient’s perception of disease activity, with respect to BASDAI. Furthermore, ASDAS has been shown to well correlate with biomarkers of cartilage [e.g. matrix metalloproteinase 3 (MMP-3) and osteocalcin] and bone turnover (e.g. C-terminal crosslinking telopeptide of type II collagen) [40]. This indicates that ASDAS may better reflect the inflammatory disease processes in SpA with comparison to BASDAI.

Even the final decision to define the most appropriate set of domains of ASDAS has not yet been taken, ASDAS-CRP is the one widely recommended [11]. ASDAS was found to be applicable also in subgroups without elevated CRP and/or peripheral swelling joints [41].

Despite the excellent psychometric properties of ASDAS for the evaluation of disease activity in axial SpA, this index is not easy to use in the everyday clinical practice. This relevant aspect has recently led to a major revision of ASDAS, in order to simplify the index [12]. Undoubtedly, the SASDAS index improves the evaluation of disease activity in daily practice and real-life conditions and, moreover, complies the recommendations of the OMERACT group [42].

In our study we have investigated the construct validity of the SASDAS in evaluating the disease activity and we have compared the internal and external responsiveness of SADSAS and ASDAS ESR/CRP and traditional BASDAI in a cohort of patients with axial SpA. Compared with conventional clinical measures of disease activity, functional and general health status, SASDAS have demonstrated adequate construct validity and was equally or more responsive to changes in disease activity than conventional composite measures.

Similarly to the original study [12], we have found a very high degree of correlation between these composite indices. The highest correlations were seen between SASDAS and ASDAS-ESR and between SASDAS and BASDAI score. Strong correlations were also found between SASDAS and ASDAS-CRP, SASDAS and BASFI and SASDAS and EQ-5D. Further, the categorization of cut-off of SASDAS versus those of both the ASDAS CRP/ESR have confirmed a significant high overall agreement.

It was recently shown that ASDAS performs better than BASDAI in evaluating disease activity in patients with AS. In particular, Lukas et al. [10] and van der Heijde et al. [11] have documented a better discriminatory capacity of ASDAS sets compared to BASDAI. Vastesaeger et al. [43], in concordance with validation of the ASDAS [11], have demonstrated that ASDAS discriminate better than BASDAI in patients with elevated CRP and was equal to BASDAI in patients with normal CRP. The ASDAS is also a highly effective measure in assessing disease activity and a great discriminatory measurement to assess the efficacy of TNF-a inhibitor in AS and undifferentiated SpA [44]. However, three other studies that have assessed the validity of BASDAI and ASDAS sets in patients with axial PsA showed conflicting results. Taylor and Harrison [39] have concluded that BASDAI correlated well with patient perception of disease activity but, was unable to discriminate well between high and low disease activity. Fernández-Sueiro et al. [45] have shown that BASDAI performed well in differentiating between patients with axial-PsA and those without axial involvement. Eder et al. [46] have demonstrated that in patients with axial-PsA, ASDAS and BASDAI scores show similar discriminative ability (from moderate to good) and correlation with different constructs of disease activity. ASDAS was not superior to BASDAI in its ability to discriminate between high and low disease activity states in axial-PsA. A confounding factor in these studies that may account for the discrepancy between the results obtained in the axial-PsA could be due to the presence of peripheral arthritis. In fact the peripheral arthritis, in these cases, may have an impact on disease activity level when it is assessed using BASDAI. This may be an advantage of ASDAS with respect to BASDAI, which is affected by peripheral involvement to various degrees, even in subjects with predominantly axial involvement [47,48].

Up-to-date, in clinical practice the decision to start or continue DMARDs or TNF-a blocking therapy in patients with axial SpA is mainly based on BASDAI response, which is solely based on the opinion of the patient. Our results showed that the simplified version of the ASDAS (SASDAS) was sensitive to improvement in patients with axial SpA receiving TNF-inhibitors, with an ES of 1.62 and a SRM of 0.88, and was more responsive than BASDAI (ES 0.93, SRM 0.52). The most efficient composite measure in detecting changes of disease activity was the ASDAS-CRP (ES 1.95; SRM 0.97), whereas the ASDAS-ESR showed an intermediate behaviour (ES 1.33; SRM 0.71).

Our results are consistent with the literature data and further support the good psychometric properties of the ASDAS. In particular, in a 46 weeks prospective, longitudinal multi-center study, Pedersen et al. [49] have investigated the construct validity and responsiveness of the ASDAS-CRP in patients with SpA treated with anti-TNF drugs. The authors demonstrated that ASDAS had higher responsiveness compared to BASDAI and CRP and thresholds for BASDAI at 20 mm or 50% improvement corresponding to an ASDAS of 1.38 and 1.95, respectively. ASDAS-CRP has demonstrated the highest responsiveness with an effect size of 2.04 and a standardized response mean of 1.45, whereas BASDAI (1.86; 1.36) and CRP (0.63; 0.70) were less responsive. Similarly, in a post hoc analysis of the randomized controller ASCEND trial, van der Heijde et al. found that ASDAS is a validated and highly discriminatory tool for the detection of significant differences between treatments for AS as well as for detecting a significant improvement from baseline with etanercept and SSZ [50].

Although comparable responses in the ASAS 20, ASAS 40 and ASAS 5/6 and the BASDAI 50 have been achieved by adalimumab, etanercept and infliximab [51–53], low to moderate levels of responsiveness were reported for the BASDAI in placebo-controlled trials and longitudinal evaluation of active drugs [54,55], in longitudinal evaluation of in-patient rehabilitation [56] or in combined spa and exercise therapy [57–61]. Mean score change for the BASDAI did not exceed 1.9 and 1.3 respectively following all physical therapy interventions within a 2 to 40-week follow-up period.

Our study was designed to test the performance of the SASDAS versus ASDAS ESR/CRP and BASDAI in the clinical routine setting, so, we aware that it presents some limitations. First, we have not correlated the composite indices with structural damage and to ensure criterion validity of the composite indices. However, this is the subject of an ongoing study. Second, our study was performed in a single centre within a relatively small catchment area. Third, our work was concentrated only to the simplified version of ASDAS-ESR. We aware tha it would be of great interest to test also the ASDAS-CRP which is currently preferred for the assessment of axial SpA. Our research agenda is currently addressed to this topic in order to improve the scientific interest. Further, it remains to be seen in future long-term analyses whether the presented SASDAS cut-offs for different stages of disease activity will show similar results.

In conclusion, in patients with axial SpA the ASDAS scoring system and SASDAS scores show similar good discriminative ability and correlation with different constructs of disease activity and health status. The SASDAS score did not improve its discriminative ability and responsiveness compared with ASDAS scoring systems. Therefore, since SASDAS is easier to calculate, it may be more practical for clinical use in patients with axial SpA.

## Additional files

Additional file 1:
**Descriptive statistic of SASDAS, ASDAS-ESR, ASDAS-CRP, BASDAI, BASFI and EQ-5D.**
Additional file 2:
**ROC plots of the change score of questionnaires.**
