# Supplementary material for: Construct validity and responsiveness of the simplified version of Ankylosing Spondylitis Disease Activity Score (SASDAS) for the evaluation of disease activity in axial spondyloarthritis
Source: Health Qual Life Outcomes. 2014 Aug 22;12:129. doi: 10.1186/s12955-014-0129-9 (PMC4243811; doi:10.1186/s12955-014-0129-9)
Supplement: Additional file 1: — Descriptive statistic of SASDAS, ASDAS-ESR, ASDAS-CRP, BASDAI, BASFI and EQ-5D. [file 12955_2014_129_MOESM1_ESM.doc]

***Additional file 1****.* Descriptive statistic of SASDAS, ASDAS-ESR, ASDAS-CRP, BASDAI, BASFI and EQ-5D

|  | **SASDAS** | **ASDAS-ESR** | **ASDAS-CRP** | **BASDAI** | **BASFI** | **EQ-5D** |
| --- | --- | --- | --- | --- | --- | --- |
| Lowest value | [0.70](cmd:SHOWXMINMAX?391) | [0.65](cmd:SHOWXMINMAX?355) | [0.23](cmd:SHOWXMINMAX?391) | [0.00](cmd:SHOWXMINMAX?391) | 0.00 | [0.30](../../../../cmd/SHOWXMINMAX%3F198) |
| Highest value | [37.20](cmd:SHOWXMINMAX?334) | [4.08](cmd:SHOWXMINMAX?321) | [4.56](cmd:SHOWXMINMAX?215) | [7.87](cmd:SHOWXMINMAX?230) | [8.80](../../../../cmd/SHOWXMINMAX%3F21) | [0.99](../../../../cmd/SHOWXMINMAX%3F117) |
| Arithmetic mean | 20.18 | 2.48 | 2.56 | 4.10 | 4.47 | 0.70 |
| 95% CI for the mean | 19.45 to 20.90 | 2.42 to 2.55 | 2.48 to 2.63 | 3.96 to 4.25 | 4.27 to 4.67 | 0.68 to 0.71 |
| Median | 20.10 | 2.49 | 2.53 | 4.00 | 4.44 | 0.68 |
| 95% CI for the median | 19.20 to 21.15 | 2.39 to 2.55 | 2.47 to 2.66 | 3.81 to 4.12 | 4.3300 to 4.4400 | 0.68 to 0.69 |
| Variance | 53.77 | 0.42 | 0.59 | 2.21 | 3.98 | 0.017 |
| Standard deviation | 7.33 | 0.65 | 0.77 | 1.48 | 1.99 | 0.13 |
| Relative standard deviation | 0.36 (36.34%) | 0.26 (26.12%) | 0.30 (30.20%) | 0.36 (36.21%) | 0.44 (44.67%) | 0.18 (18.82%) |
| Standard error of the mean | 0.36 | 0.032 | 0.038 | 0.074 | 0.100 | 0.006 |
| Coefficient of Skewness | -0.10 (P=0.38) | -0.13 (P=0.28) | -0.20 (P=0.09) | -0.036 (P=0.76) | 0.023 (P=0.84) | -0.181(P=0.12) |
| Coefficient of Kurtosis | -0.31 (P=0.18) | -0.16 (P=0.53) | 0.048 (P=0.75) | 0.034 (P=0.79) | -0.383(P=0.06) | -0.075 (P=0.83) |
| Kolmogorov-Smirnov test for Normal distribution | accept Normality (P=0.48) | accept Normality (P=0.99) | accept Normality (P=0.96) | accept Normality (P=0.13) | reject  Normality (P=0.03) | reject  Normality (P=0.006) |
