# Supplementary material for: Construct validity and responsiveness of the simplified version of Ankylosing Spondylitis Disease Activity Score (SASDAS) for the evaluation of disease activity in axial spondyloarthritis
Source: Health Qual Life Outcomes. 2014 Aug 22;12:129. doi: 10.1186/s12955-014-0129-9 (PMC4243811; doi:10.1186/s12955-014-0129-9)
Supplement: Additional file 2: — ROC plots of the change score of questionnaires. [file 12955_2014_129_MOESM2_ESM.doc]

***Additional file 2***. ROC plots of the change score of questionnaires.

|  | **AUC** | **Standard**  **Error** | **95% CI** |
| --- | --- | --- | --- |
| Change score of SASDAS | 0.870 | 0.031 | 0.808 to 0.932 |
| Change score of ASDAS-ESR | 0.804 | 0.037 | 0.721 to 0.877 |
| Change score of ASDAS-CRP | 0.882 | 0.028 | 0.826 to 0.937 |
| Change score of BASDAI | 0.787 | 0.041 | 0.704 to 0.868 |
